# Multivariate assessment of morpho-biochemical and bioactive diversity in *Syzygium cumini* (L.) Skeels for the selection of superior genotype and breeding applications

**DOI:** 10.1186/s12870-025-06977-x

**Published:** 2025-07-26

**Authors:** Ajay Kumar, Kanhaiya Singh, Jai Prakash, Amit Kumar Goswami, Vishaw Bandhu Patel, Aditya Dnyaneshwar Ingole, Bhupendra Sagore, Gyan Prakash Mishra, Virendra Singh Rana, Rakesh Bhardwaj, Amit Kumar Singh

**Affiliations:** 1 Division of Fruits and Horticultural Technology, ICAR-Indian Agricultural Research Institute, New Delhi, 110012 India; 2 Horticultural Science Division, ICAR-Krishi Anusandhan Bhawan-II, New Delhi, 110012 India; 3 Division of Genetics, ICAR-Indian Agricultural Research Institute, New Delhi, 110012 India; 4 Division of Agricultural Chemicals, ICAR-Indian Agricultural Research Institute, New Delhi, 110012 India; 5 ICAR-National Bureau of Plant Genetic Resources, New Delhi, 110012 India

**Keywords:** Biochemical, Bioactive, Diversity, Genetic resource, Morphology, *Syzygium cumini*

## Abstract

**Background jamun:**

[*Syzygium cumini* (L.) Skeels] is an underutilized fruit crop in India, despite having numerous medicinal and nutritional benefits. Although the Indian subcontinent harbours a vast natural population of *Jamun*, limited knowledge exists regarding superior genotypes. This study evaluates the diversity among *Jamun* genotypes using morpho-biochemical and bioactive traits to facilitate superior genotype selection and commercial breeding applications.

**Results:**

Twenty-seven characteristics were studied to identify superior genotypes from a seedling-origin *Jamun* population. Significant variation was recorded in fruit and seed-related traits. The pulp-to-seed ratio exhibited the highest variation. Predominant fruit shape was oblong, with variation in fruit stalk end, fruit color and pulp color. Biochemical traits, such as TSS, TSS: acid ratio, total sugar and non-reducing sugars, showed significant variability. Similarly, substantial variation was observed in bioactive traits, including ascorbic acid, total phenolic content, total flavonoid content and antioxidant activity. Genotypes PCJ-9 and PCJ-17 exhibited higher fruit weight, pulp weight, pulp content, pulp-to-seed ratio, TSS, vitamin-C, with lower phenolic content and medium values of total flavonoid and antioxidant activity. Six genotypes (PCJ-15, PCJ-30, PCJ-22, PCJ-3, PCJ-1 and PCJ-16) showed high TPC, TFC, antioxidant and seed content, making them suitable for breeding bioactive-rich cultivars and functional food development. Pearson correlation analysis evaluated a significant positive correlation between fruit weight and pulp weight, fruit size and fruit width. PCA analysis identified PC1 and PC2 as the most important components. Heatmap clustering analysis emphasized the relationship between fruit morph-biochemical and bioactive characteristics.

**Conclusions:**

The presented study offers a novel and comprehensive assessment of diversity among seedling-origin *Jamun* genotypes based on morpho-biochemical and bioactive traits. The identified superior genotypes present promising candidates for cultivar development and direct cultivation, contributing to improved *Jamun* productivity and functional food value.

**Supplementary Information:**

The online version contains supplementary material available at 10.1186/s12870-025-06977-x.

## Introduction

Indian blackberry [*Syzygium cumini* (L.) Skeels], also known as *Jamun*,* jambul*, *jambolan*, black plum, danson plum, Malabar plum, Portuguese plum and *Java plum*, belongs to the Myrtaceae family [1, 2]. *Jamun* is a potential underutilized minor fruit tree thriving in tropical and subtropical regions. Indigenous to India, it has naturalized in Asian subcontinent, East African countries, Southern America and is also grown in Hawaii and Florida [3]. It grows well in neglected and marshy lands where other fruit plants fail to thrive [4]. *Jamun* is a large, densely foliaceous evergreen tree, reaching up to 100 ft, with significant morphological and fruiting diversity [5, 6]. The oval, fleshy, succulent, single-seeded berry-like fruits and have a purple mesocarp with a purplish-black pericarp. The fruits are eaten fresh and processed into juice, jam, jelly, ice cream, wine, vinegar, pudding and other value-added products [7, 8].

*Jamun* is well recognized for its substantial medicinal importance. *Jamun* fruits and various plant parts have been traditionally used in various medicinal systems to treat several human maladies [9]. *Jamun* is particularly valued for its anti-diabetic properties. Various plant parts possess hypoglycemic, anti-anemic, antioxidant, antiallergic, hepatoprotective, antibacterial, hypolipidemic, antipyretic and anti-inflammatory bioactivities [10]. These nutritional and medicinal properties have made jamun more relevant in the 21 st century [11].

Despite being native to India and possessing significant potential, genetic enhancement of jamun remains minimal [12]. Genetic variation plays a crucial role in the adaptation and climate resilience of long-lived tree species. Populations with great genetic diversity are better able to endure environmental difficulties, habitat fragmentation and population reduction, increasing their chances of survival [13].

However, its growing therapeutic potential underscores the need for intensive cultivar development strategies. Most jamun trees grown in India are seedling origin, exhibiting tremendous variation in morphological and physicochemical characters, including fruit size, shape, pulp percent, TSS and acidity [14–16]. Documenting these variations is crucial for identifying elite clones with superior yield and quality traits.

Previous studies primarily focused on morphological and biochemical diversity in jamun, recommending these traits for germplasm characterization and evaluation [14, 15, 17–19]. However, a comprehensive assessment integrating morphological, biochemical and bioactive traits remains largely unexplored. Moreover, the absence of a well-defined standard variety continues to be a significant constraint in the expansion and commercialization of jamun cultivation in India [14, 15]. Thus, a comprehensive characterization of fruits’ morpho-biochemical and bioactive characteristics is critical for understanding the extent of genetic variability and selecting promising genotypes. Multivariate approaches such as correlation matrix analysis, principal component analysis and clustering using heat map, have been proven valuable in characterizing and selecting superior genotypes in fruit crops such as almond, walnut, fig, pomegranate etc [21–24].

The Indian *Syzygium cumini* germplasm represents an underutilized yet genetically diverse resource with immense potential for genetic improvement. Therefore, the present study aims to provide a novel and integrative evaluation of morpho-biochemical and bioactive diversity in seedling-origin *Jamun* genotypes, to identify promising selections possessing desirable commercial traits for both fresh consumption and functional food applications.

## Materials and methods

### Plant material

**Fig. 1:**
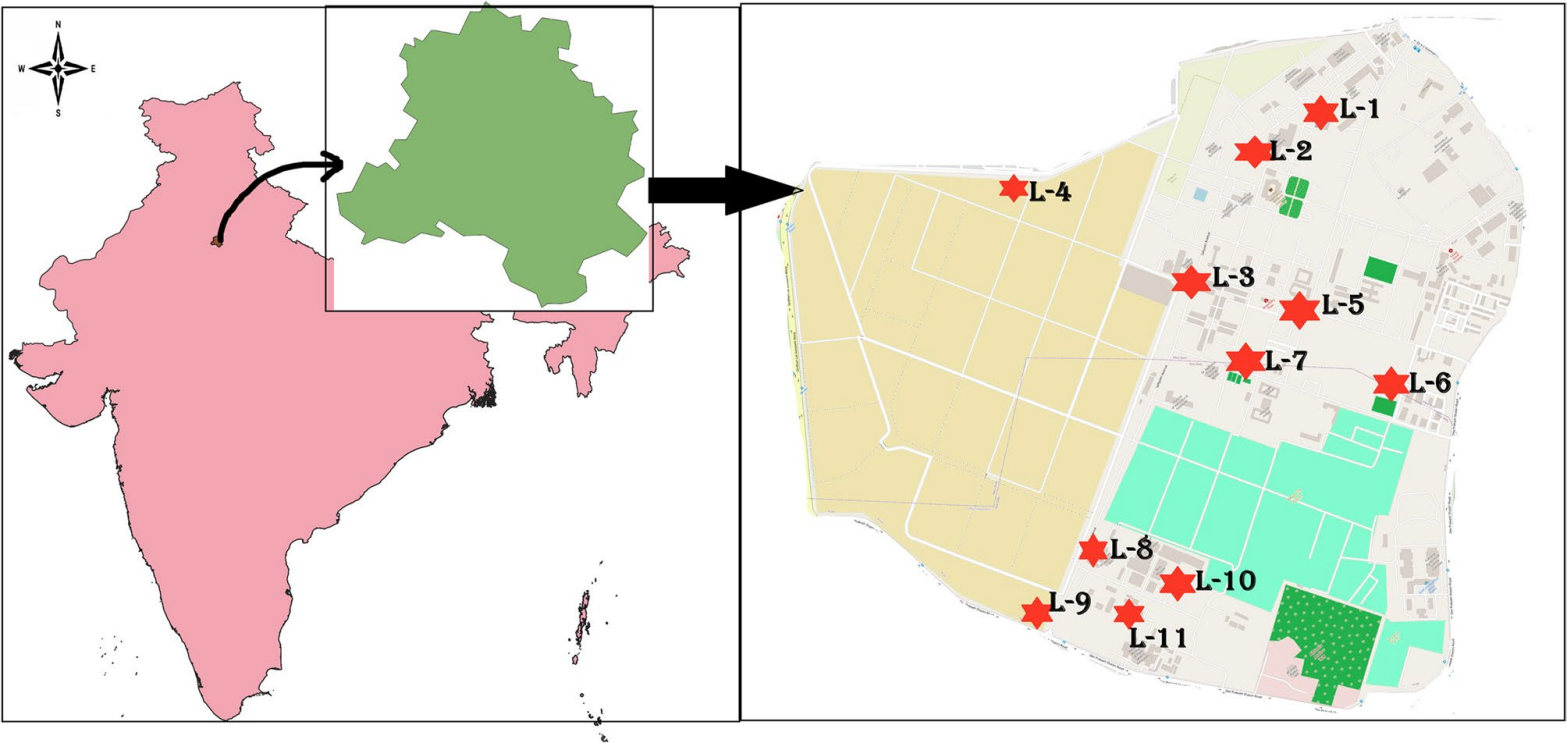
Map representing sample collection sites of *Syzygium cumini* (L.) Skeels. Red stars labeled L-1 to L-11 indicate distinct sampling sites where individual genotypes were collected

Based on a preliminary study, 30 diverse *Jamun* genotypes were selected from a large natural population of seedlings origin spread across the ICAR-Indian Agricultural Research Institute, New Delhi (Table S1). The location (28.40 N, 77.10E) is a hot semi-arid, with an average yearly precipitation of 797.3 mm, 39 rainy days, and a mean temperature of 34 ℃. Sampling was conducted at 11 different locations (Fig. 1), each sample collected 20 m apart. A standard check, CISH J-37, was added in this study to facilitate the comparison of morpho-biochemical traits. This cultivar was obtained from ICAR-Central Institute of Subtropical Horticulture (CISH), Lucknow, Uttar Pradesh, India. All genotypes were evaluated for morpho-biochemical and bioactive characteristics over two consecutive calendar years (2023–2024).

### Morphological characters

Sixteen morphological traits were examined, among them, eleven were quantitative and five were qualitative. From each genotype, 40 mature fruits were randomly harvested to record data. Fruit length, fruit width, seed length and seed width were measured using digital Vernier Calipers and expressed in cm. Fruit weight, seed weight and pulp weight were measured using an electronic balance with 0.01 g precision. Five qualitative characteristics, namely fruit shape, fruit apex, fruit stalk end, fruit color and fruit pulp color, were coded using standard *Jamun* descriptors [25].

### Biochemical characters

#### Total soluble solids (TSS)

TSS of *Jamun* fruits was estimated by a drop of juice squeezed into the prism of a portable refractometer at 20 °C. The degree Brix (°B) was used to measure the light refraction [26].

#### Titratable acidity (TA)

Crushed fruit pulp weighing two grams was blended with distilled water and centrifuged. To calculate the titratable acidity, that is represented as a percentage (%) of citric acid, titration of homogenate against 0.1 N NaOH was performed [26].

#### Total sugar

The method developed by Lane and Eynon [27] and adopted by [17] was used to estimate the total, reducing and non-reducing sugar content in *Jamun*. A mixture of distilled water, potassium oxalate and lead acetate were prepared using 25 mL of *Jamun* fruit juice and diluted to make up a final volume of 250 mL. Total sugar content was determined by titrating 50 mL of this sample, after inversion with HCl and neutralizing with NaOH using Fehling’s solution and methylene blue as an indicator. Fehling’s factor, representing the amount of reducing sugar (g) that corresponds to 1 mL of Fehling’s solution, was determined by standardizing the solution with a known glucose concentration and used in the sugar calculations. Reducing sugars were quantified by filtration, titration with Fehling’s solutions and precipitations of contaminants using lead acetate and potassium oxalate. The reducing sugar percentage was subtracted from the overall sugar percentage, and the result was multiplied by 0.95 to determine the non-reducing sugar content.

$$\mathrm{Total}\;\mathrm{sugar}\;\left(\%\right)=\frac{\mathrm{Fehling}'\mathrm s\;\mathrm{factor}\times\mathrm{Dilution}\;\mathrm{Factor}\times100}{\mathrm{Titre}\;\mathrm{volume}\;\times\mathrm{sample}\;\mathrm{mass}}$$

$$\mathrm{Reducing}\;\mathrm{sugars}\;\left(\%\right)=\frac{\mathrm{Fehling}'\mathrm s\;\mathrm{factor}\times\mathrm{Dilution}\;\mathrm{Factor}\times100\times100}{\mathrm{Titre}\;\mathrm{volume}\times\mathrm{sample}\;\mathrm{mass}\times50}$$

Non-reducing sugar (%) = (Total sugar content–reducing sugar content) × 0.95.

#### Bioactive characters

The following bioactive assays were conducted using 20 randomly selected mature fruits per genotype.

#### Ascorbic acid

The amount of ascorbic acid in *Jamun* fruit was estimated by employing a visual titration technique with dye 2,6-dichlorophenol indophenols [28, 29]. The results were expressed as mg of ascorbic acid per 100 g of fresh weight (FW), calculated using the following formula.

$$\:\text{A}\text{s}\text{c}\text{o}\text{r}\text{b}\text{i}\text{c}\:\text{a}\text{c}\text{i}\text{d}\:(\text{m}\text{g}/100\:\text{g})=\frac{\text{T}\text{i}\text{t}\text{r}\text{e}\:\times\:\text{D}\text{y}\text{e}\:\text{f}\text{a}\text{c}\text{t}\text{o}\text{r}\:\times\:\text{V}\times\:100}{\text{A}\text{l}\text{i}\text{q}\text{u}\text{o}\text{t}\:\text{o}\text{f}\:\text{e}\text{x}\text{t}\text{r}\text{a}\text{c}\text{t}\:\times\:\text{m}}$$

where Dye factor = 0.5/titre value (mg/mL)

Titre: Volume of dye used in titration (mL)

V: Total volume of extract prepared (mL)

Aliquot of extract: Volume of extract used for titration (mL)

m: Weight of fruit sample (g)

100: Factor to express the result per 100 g FW

#### Total phenolic content (TPC)

The total phenolic content (TPC) of *Jamun* fruits was analysed employing the Folin-Ciocalteau reagent method [30]. In brief, 2 g of pulp was crushed with 20 mL of 80% methanol and centrifuged at 10,000 × g for 20 min at 4 °C. The obtained supernatant was analysed as Gallic acid equivalent (GAE) in milligrams per 100 g of fresh weight (FW).

#### Total flavonoid content (TFC)

According to Zhishen et al. [31] methodology, a spectrophotometer was used to measure the total flavonoid content. In a 10 ml volumetric flask, 1 ml of the sample extract and 0.3 ml of NaOH were added and the volume was raised to 4 ml using distilled water. Then, 10% AlCl_3_•6H_2_O (0.3 ml) was added. After adding 2 milliliters of 1 N NaOH, the solution was diluted with distilled water to reach a volume of 10 milliliters. Immediately, the absorbance was measured with a spectrophotometer at 510 nm. The following formula was used to determine the flavonoid concentration and represent it as quercetin equivalents (QE).

$$\mathrm{Total}\;\mathrm{flavonoid}\;\mathrm{content}=\frac{\mathrm{Optical}\;\mathrm{density}\;\times\mathrm{volume}\;\mathrm{made}\;\mathrm{up}\times\mathrm{dilution}\;\mathrm{factor}\times0.1}{\mathrm{Factor}\;\mathrm{for}\;\mathrm{QE}(0.001)\times\mathrm{Weight}\;\mathrm{of}\;\mathrm{sample}\times\mathrm{Aliquot}}$$

##### Total antioxidant activity

Twenty-five milligrams of DPPH(2,2-diphenyl-1-picrylhydrazyl) was dissolved in 100 ml of methanol to prepare the stock solution, and the absorbance of 0.70 ± 0.01 was recorded at 517 nm, in accordance with the procedure established by Brand-Williams et al. [32] and [33] with a few minor adjustments to determine the DPPH inhibition %. Homogenizing 2 g of *Jamun* fruits was done with 10 mL of methanol, centrifuged at 10,000 x g for 20 min. The supernatant of 100 µl was mixed with 3.9 mL of the DPPH stock solution. The mixture was incubated for two hours in the dark, and the absorbance of the mixture was measured at 517 nm. The DPPH scavenging activity was estimated by comparing the absorbance to a reagent blank.

$$\mathrm{DPPH}\;\mathrm{Inhibition}\;(\%)=\left\{\frac{\mathrm{Ac}-\mathrm{As}}{\mathrm{Ac}}\right\}\times100$$

Where, Ac = Absorbance (optical density) of DPPH.

As = Absorbance of sample.

#### Instrumentation

Fruit dimensions were measured with digital calipers (Mitutoyo, Japan) and weights with a precision balance (Aczet, India; 0.01 g). TSS was measured using a refractometer (Atago, Japan). A centrifuge (Sigma 3–18 K, Germany) was used for sample preparation and a UV-spectrophotometer (UV 5704SS, ECIL, India) was used for TPC, TFC and DPPH assays.

#### Statistical analysis

SAS software (Version 9.0) [34] was used to analyse variation across all characters. Descriptive statistics, which include mean, minimum, maximum, standard deviation (SD), and coefficient of variation (CV%), were computed for all the recorded traits. The frequency and percentage distribution of qualitative attributes were analyzed. The Tukey test was employed to compare means. Pearson (r) correlation coefficients (r) were calculated by SPSS software to assess relationships between observed variables. Principal Component Analysis (PCA) was conducted to identify key patterns in the dataset. Additionally, a heat map was generated using Ward’s clustering method and Euclidean distance coefficients to visualise genotype associations.

## Results

### Morpho-biochemical characteristics

**Fig. 2:**
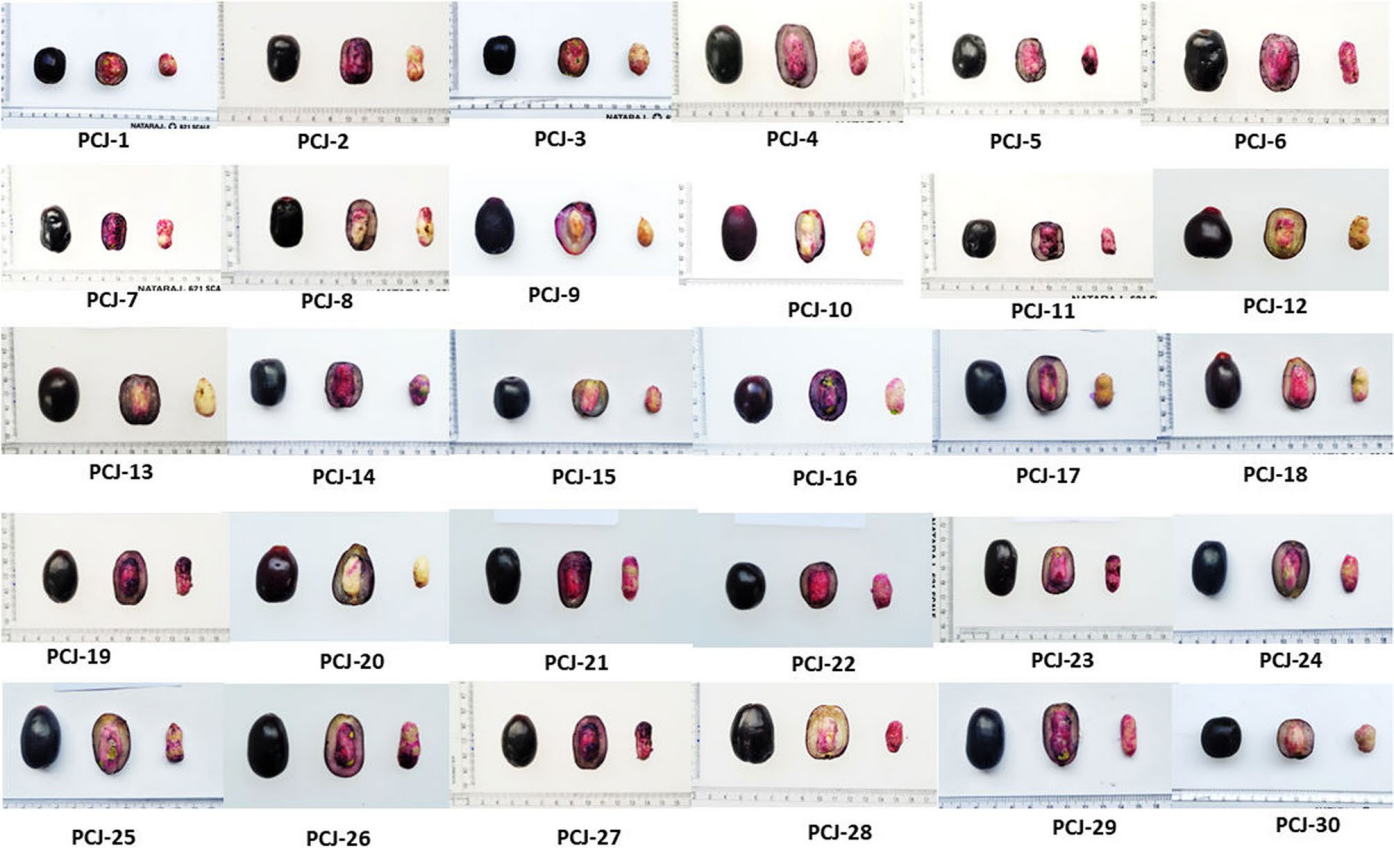
Representative fruits of some diverse genotypes of *Syzygium cumini* (L.) Skeels

Analysis of morphological traits is an initial step of the evaluation of genetic diversity within a plant species. Variations in morpho-logical traits among 31 jamun genotypes revealed significant differences in most traits (Table 1). The studied *Jamun* genotypes displayed remarkable variability across the examined pomological and biochemical characteristics. Some representative fruits of diverse genotypes are shown in Fig. 2. The highest coefficients of variation (CVs) were observed for fruit pulp color (79.24%), pulp-to-seed ratio (43.94%) and pulp weight (40.77%). In contrast, the lowest CV value was observed for pulp content (6.49%). Among 23 measured morpho-biochemical traits, 12 traits exhibited CVs higher than 20%, showing a high level of diversity among the genotypes (Table 1).

**Table 1:** Descriptive statistics for the morphological and biochemical characters in the studied genotypes of *Syzygium cumini* (L.) Skeels genotypes

No	Trait	Unit	Min	Max	Mean	SD	CV (%)
V1	Fruit weight	g	5.05	22.47	9.63	3.68	38.25
V2	Fruit length	cm	2.33	4.14	2.98	0.43	14.54
V3	Fruit width	cm	1.59	2.87	2.04	0.28	13.65
V4	Fruit size	cm^2^	4.28	10.19	6.11	1.56	25.53
V5	Pulp weight	g	3.51	17.50	7.56	3.08	40.77
V6	Pulp content	%	69.43	94.49	78.47	5.09	6.49
V7	Seed length	cm	1.38	2.61	2.11	0.28	13.14
V8	Seed width	cm	0.73	1.38	0.93	0.17	18.52
V9	Seed weight	g	1.19	2.89	1.95	0.52	26.83
V10	Seed content	%	8.01	30.57	21.62	4.85	22.42
V11	Pulp to seed ratio	Ratio	2.29	11.67	4.04	1.77	43.94
V12	Fruit shape	Code	3.00	9.00	5.32	2.07	38.93
V13	Fruit apex	Code	3.00	7.00	4.68	1.64	35.08
V14	Fruit stalk	Code	3.00	7.00	5.58	1.48	26.49
V15	Fruit skin color	Code	1.00	3.00	2.10	0.87	41.49
V16	Fruit pulp color	Code	1.00	9.00	5.13	4.06	79.24
V17	Total soluble solids	%	10.49	16.91	13.04	1.57	12.02
V18	Titratable acidity	%	0.81	1.08	1.01	0.06	5.65
V19	Total soluble solids to titratable acidity ratio	Ratio	10.08	19.76	13.06	2.22	16.97
V20	Total sugar	%	8.32	16.87	12.03	1.83	15.24
V21	Reducing sugar	%	7.90	16.06	11.57	1.76	15.24
V22	Non-reducing sugar	%	0.11	1.40	0.46	0.28	60.11
V23	Sugar to acid ratio	Ratio	7.81	18.68	12.01	2.31	19.22
V24	Ascorbic acid	mg	23.62	43.34	30.18	4.20	13.92
V25	Total phenolic content	mg GAE/100 g	71.40	184.01	116.28	32.44	27.90
V26	Total flavonoid content	mg QE/100 g	52.63	102.11	69.87	11.60	16.59
V27	Antioxidant activity	%	32.58	93.55	57.60	21.76	37.77

*Max* Maximum, *Min* Minimum, *SD* Standard Deviation, *CV* Coefficient of Variation

**Fig. 3:**
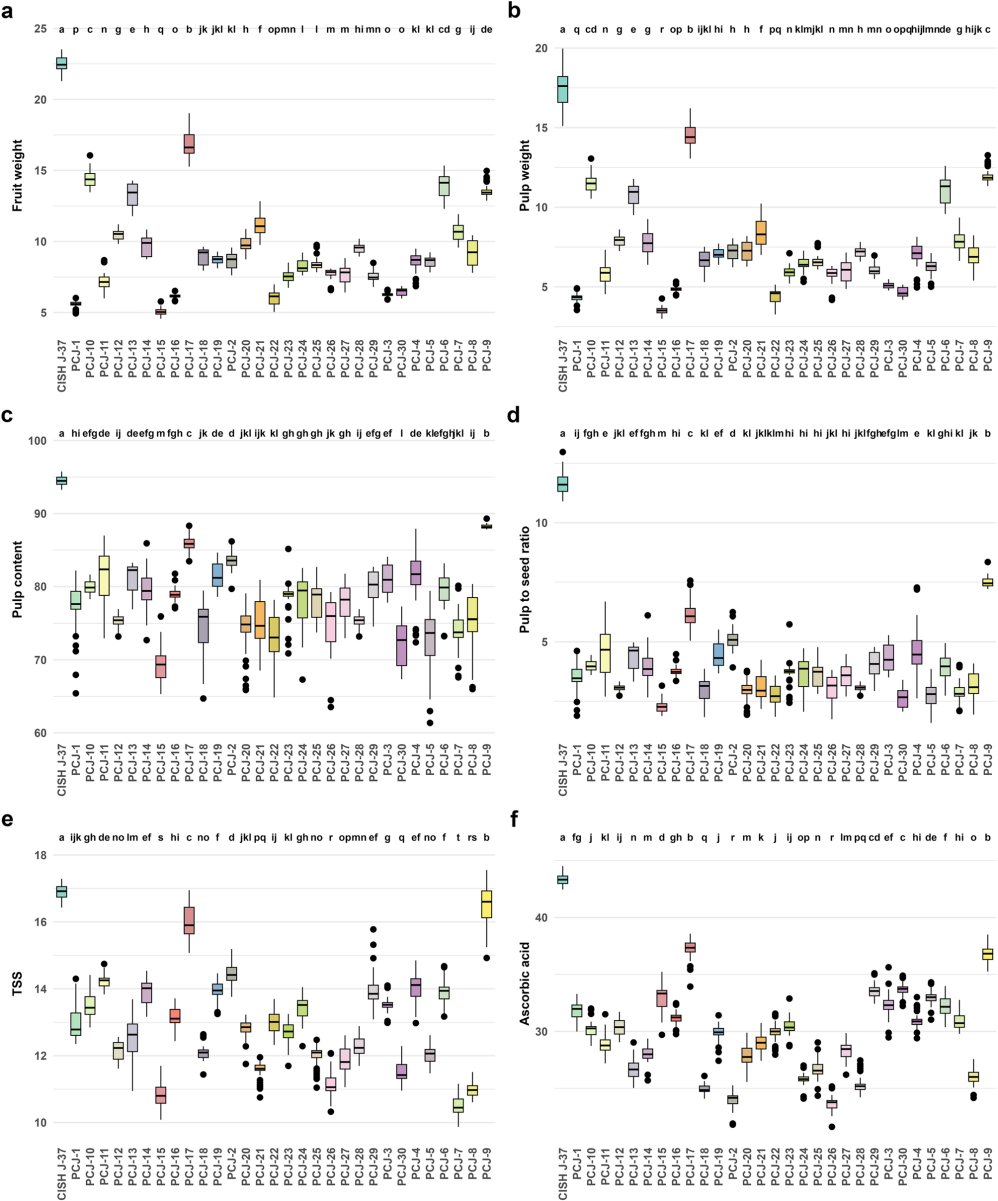
Boxplot analysis of tukey test based on fruit weight (**a**), pulp weight (**b**), pulp content (**c**), pulp-to-seed ratio (**d**), total soluble solids (**e**) and ascorbic acid content (**f**) of *Syzygium cumini* (L.) Skeels genotypes

Fruit mass and pulp-related characters such as fruit weight, fruit length, fruit width, fruit size, pulp weight and pulp content are very important traits for consumer preference, therefore, these are very valuable for the selection of superior genotypes. Significant variation was recorded in fruit and seed-related traits. Fruit weight varied from 5.05 to 22.47 g and pulp weight from 3.51 to 17.50 g. These variations suggest promising potential for selecting genotypes with superior fruit size and pulp yield. The highest fruit weight was observed in CISH J-37 (22.46 g) followed by PCJ-17 (16.89 g), PCJ-10 (14.43 g), PCJ-6 (13.99 g) and PCJ-9 (13.57 g). The lowest value of this trait was recorded in PCJ-5 (5.04 g) (Fig. 3a). Fruit length and fruit width showed more stability, ranging from 2.33 cm to 4.14 cm and 1.59 cm to 2.87 cm, respectively. Fruit size showed moderate variability, ranging from 4.28 cm² to 10.19 cm². The pulp weight varied from 3.51 to 17.50 g and pulp content ranged from 69.43 to 94.49%. In both of these traits, CISH J-37, PCJ-17 and PCJ-9 recorded the highest values (Fig. 3b and c). Seed-related traits also revealed significant variability. Seed weight ranged from 1.19 g to 2.89 g, seed content varied from 8.01 to 30.57%, while seed length varied from 1.38 to 2.61% and seed width 0.73 to 1.38 cm. The pulp-to-seed ratio, an important indicator of fruit edibility, exhibited the greatest variation, ranging from 2.29 to 11.67 (Table 1). The highest pulp-to-seed ratio was found in CISH J-37, PCJ-17 and PCJ-9, while the lowest was in PCJ-15 (Fig. 3d).

In addition, frequency distribution analysis of five qualitative pomological traits also showed considerable diversity. The fruit shape was predominantly oblong (35.48%), followed by ovate (32.26%), elliptic (22.58%) and globose (9.68%). The fruit apex was predominantly flat (41.94%), while 32.26% of genotypes showed a depressed apex and 25.81% a rounded apex. The fruit stalk end showed notable variation, with a majority of genotypes having a depressed stalk end (45.16%), followed by flattened (38.71%) and nipple-shaped (16.13%). The fruit color ranged from purple-black (41.94%) to purple-red (32.26%) and dark purple (25.81%). The fruit pulp color was predominantly purple-white (51.61%), while the remaining genotypes had cream-white pulp (48.39%) (Table 2).

**Table 2:** Frequency distribution for the measured qualitative morphological characteristics in *Syzygium cumini* (L.) Skeels genotypes

Characteristics	Frequency (No. of genotypes)			
Fruit Shape	Oblong (11)	Ovate (10)	Elliptic (7)	Globose (3)
Fruit Apex	Flat (13)	Depressed (10)	Round (8)	**-**
Fruit Stalk End	Depressed (14)	Flattened (12)	Nipple shape (5)	**-**
Fruit Colour	Purple black (13)	Purple red (10)	Dark purple (8)	**-**
Fruit Pulp Colour	Purple white (16)	Cream white (15)	-	**-**

Significant differences were observed in biochemical traits, which influence fruit sweetness, acidity balance and nutritional content. The highest value was recorded for non-reducing sugar (60.11%), while the lowest was for titratable acidity (5.65%). Total soluble solids (TSS), a key character of fruit sweetness, ranged from 10.49 to 16.91 °Brix and the highest TSS was found in CISH J-37, PCJ-17 and PCJ-9 genotypes (Fig. 3e). Titratable acidity, which contributes to fruit tartness, was relatively stable (0.81–1.08%). The TSS: acid, an indicator of fruit taste balance, exhibited moderate values, ranging from 10.08 to 19.76. Total sugar content ranged from 8.32 to 16.87%, with reducing sugar (7.90–16.06%) forming the major fraction. Non-reducing sugar showed varied from 0.11 to 1.40%. These parameters indicate differences in sugar composition among genotypes (Table 1).

### Bioactive characteristics

Bioactive variables demonstrated a wide array of diversity. Ascorbic acid ranged from 23.62 to 43.34 mg/100 g and the highest values were found in CISH J-37, PCJ-17 and PCJ-9. Total phenolic content (TPC) and total flavonoid content (TFC), indicators of antioxidant potential, showed substantial variation from 71.40 to 184.01 mg GAE/100 and 52.63 to 102.11 mg QE/100, respectively. The DPPH assay revealed antioxidant activity, with 32.58 to 93.55% inhibition and a high CV of 37.77% was observed for this trait (Table 1). The high variability observed in ascorbic acid, total phenolic content, total flavonoid content and antioxidant activity. These highlight the potential for selecting genotypes with superior nutritional and health-promoting properties, making them valuable for breeding programs, targeting bioactive-rich cultivar development and preparation of functional foods.

### Correlation matrix analysis

**Fig. 4:**
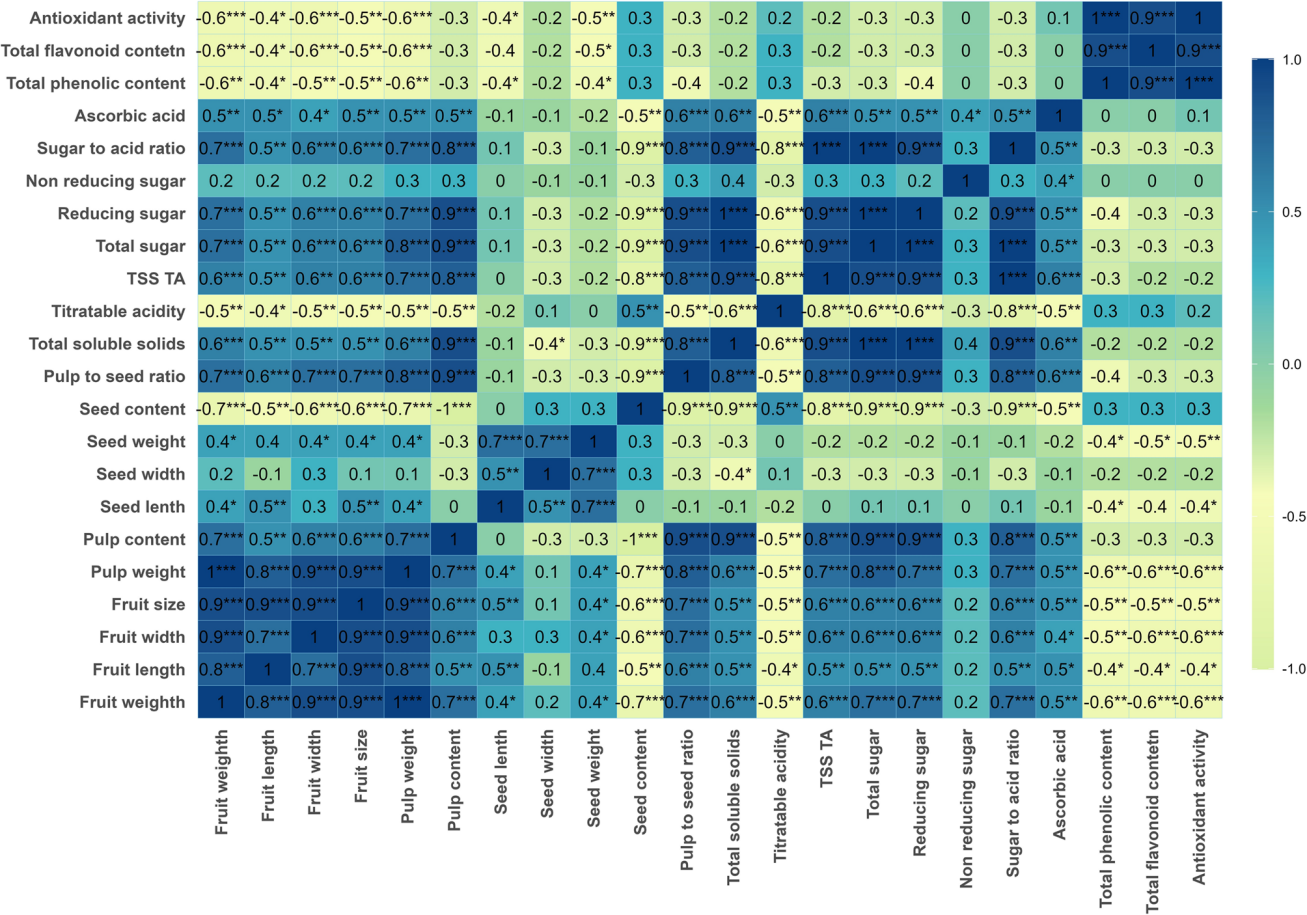
Simple correlations among the quantitative variables utilized in the studied genotypes of *Syzygium cumini* (L.) Skeels. Stronger positive correlations are shown in deeper blue, while negative correlations are indicated by yellow-green. Significance levels are denoted by asterisks (*p* < 0.05, *p* < 0.01, *p* < 0.001)

Significant and positive association between most of the quantitative morpho-biochemical and bioactive variables of jamun genotypes is observed in this study (Table S2). Based on the Pearson correlation coefficients between the morpho-biochemical and bioactive features of the fruit, a heatmap was created (Fig. 4) and a detailed correlation matrix is given in Table S2. The pale green/yellow indicates weak or no connection (–0.30 < *r* < + 0.30), dark green indicates strong negative correlation (*r* < − 0.70) and dark blue shows strong positive correlation (*r* > 0.70). Color intensity reveals the strength and direction of the link. Statistical significance is indicated by asterisks (*p < 0.05, **p < 0.01, ***p < 0.001).

Notably, fruit weight exhibited significant positive correlation with pulp weight (*r* = 0.99**), fruit size (*r* = 0.93**) and fruit width (*r* = 0.92**), as well as a strong correlation with fruit length (*r* = 0.80**). Pulp content was strongly correlated with the pulp-to-seed ratio (*r* = 0.93**), demonstrating its significance in determining the edible fraction of the fruit. Seed contents negatively correlated with fruit weight (*r* = −0.65**) and pulp content (*r* = −0.72**), indicating that larger fruits tend to have less seed mass relative to their pulp.

Among the biochemical characteristics, TSS was positively correlated with total sugar (*r* = 0.97**), reducing sugar (*r* = 0.95**) and the sugar-to-acid ratio (*r* = 0.93**). On the other hand, titratable acidity exhibited a negative correlation with TSS (*r* = −0.61**), highlighting its role in influencing fruit sweetness and flavor balance. Moreover, a positive correlation was noted between TSS: acid ratio and sugar: acid (*r* = 0.98**), showing its significance in fruit sensory qualities. A strong positive correlation was observed between ascorbic acid content and sugar-to-acid ratio (*r* = 0.54*) that revealed genotypes with a greater ratio of sugars and acids may contain more vitamin C. TPC demonstrated a negative connection with fruit weight (*r* = −0.55**) and TSS (*r* = −0.20**), indicating a potential trade-off between fruit size, sweetness and antioxidant properties. In a similar trend, TFC and antioxidant activity were also negatively correlated with fruit weight (*r* = −0.611** and *r* = −0.578**, respectively) and pulp weight (*r* = −0.597** and *r* = −0.568**, respectively). TFC indicated a strong positive correlation with TPC (*r* = 0.938**) and antioxidant activity (*r* = 0.944**). Furthermore, it demonstrates the biochemical coherence between flavonoids and overall antioxidant potential in *Jamun* genotypes.

### Principal component analysis (PCA)

**Fig. 5:**
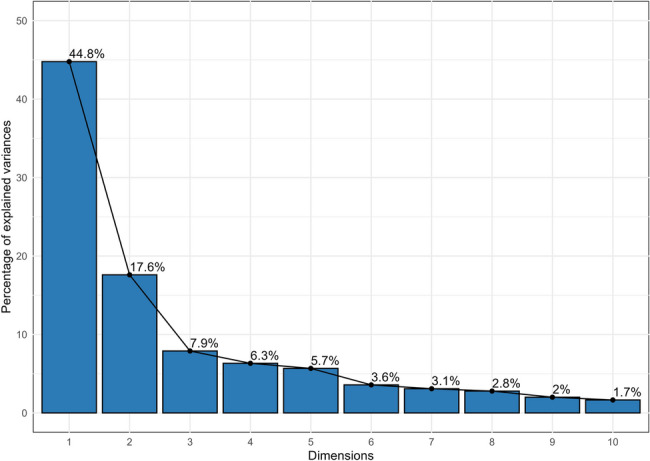
Scree plot, variance explained by principal components derived from morpho-biochemical and bioactive traits of *Syzygium cumini* (L.) Skeels genotypes. PC1 and PC2 contributed 44.8% and 17.6% of total variance, respectively

PCA is an efficient multivariate approach to clustering associated variables into principal components, reducing the data complexity. Each component contributes a proportion of the overall variance, with the primary one providing the most variance and the subsequent components accounting for the remaining variance. Table 3 displays the outcomes and the variance shared by each principal component. The first five principal components (PCs) explained 82.29% of the overall variation (Fig. 5). Bold values represent the most influential variables for every PC (Eigenvalues ≥ 0.58). PC1 explained 44.78% of the overall variance, with the largest percentages from fruit weight (0.89), fruit width (0.83), fruit size (0.85), pulp weight (0.93), pulp content (0.88), TSS (0.85), TSS: acid ratio (0.86), total sugar (0.92), reducing sugars (0.91) and sugar to acid ratio (0.90). A variance of 17.60% (PC2) was mainly contributed by seed length (−0.65), seed width (−0.66) and seed weight (−0.89) and antioxidant contributing traits such as TPC (0.58), TFC (0.62) and antioxidant activity (0.64). These findings suggest that fruit mass-related, seed-related, biochemical and antioxidant potential are key to classifying *Jamun* genotypes. PC3, which accounted for 7.90% of the total variance, that highly impacted by fruit shape (0.78), indicating that morphological features may serve as an additional distinguishing characteristic among genotypes. PC4 explained 6.33% of the total available variance, primarily related to fruit apex shape (−0.61). Moreover, PC5 shared variance (5.68%) notable contribution from fruit stalk (0.52) and bioactive traits, highlighting a supplementary role of bioactive profile in distinguishing the *Jamun* genotypes.

**Table 3:** Eigenvalues of the principal component axes from the PCA of morpho-biochemical and bioactive characters in the studied genotypes of *Syzygium cumini *(L.) Skeels

**Traits**	**Component**				
	**1**	**2**	**3**	**4**	**5**
Fruit weight	0.89**	-0.33	0.18	-0.03	0.14
Fruit length	0.73**	-0.27	0.4	-0.09	0.13
Fruit width	0.83**	-0.35	0.04	0.06	0.19
Fruit size	0.85**	-0.34	0.25	-0.03	0.21
Pulp weight	0.93**	-0.26	0.14	-0.04	0.15
Pulp content	0.88**	0.33	-0.09	-0.19	0.01
Seed length	0.26	-0.65**	0.01	0.11	0.34
Seed width	-0.11	-0.66**	-0.15	0.33	0.44
Seed weight	0.09	-0.89**	0.11	0.06	0.29
Seed content	-0.87**	-0.34	0.12	0.2	-0.02
Pulp to seed ratio	0.88**	0.28	0.06	-0.07	-0.07
Fruit shape	-0.17	0.06	0.78**	0.11	-0.23
Fruit apex	-0.22	0.06	0.4	-0.61**	0.27
Fruit stalk	-0.32	0.35	-0.21	-0.41	0.52
Fruit skin color	-0.44	0.18	-0.35	0.44	0.21
Fruit pulp color	-0.1	0.49	-0.49	0.05	0.41
Total soluble solids	0.85**	0.46	-0.02	-0.03	-0.04
Titratable acidity	-0.67**	-0.17	0.17	-0.39	-0.1
Total soluble solids to titratable acidity ratio	0.86**	0.39	-0.08	0.14	0.02
Total sugar	0.92**	0.31	-0.06	-0.05	0
Reducing sugar	0.91**	0.3	-0.09	-0.14	0.03
Non-reducing sugar	0.33	0.14	0.16	0.55	-0.22
Sugar to acid ratio	0.9**	0.29	-0.12	0.1	0.04
Ascorbic acid	0.56**	0.32	0.37	0.41	0.13
Total phenolic content	-0.55	0.58**	0.4	0.11	0.35
Total flavonoid content	-0.55	0.62**	0.28	0.16	0.32
Antioxidant activity	-0.54	0.64**	0.41	0.13	0.29
Eigenvalue	12.09	4.75	2.13	1.71	1.53
*% of Variance *	44.78	17.6	7.9	6.33	5.68
*Cumulative %*	44.78	62.38	70.28	76.61	82.29

** Eigenvalues are significant ≥ 0.58

### Bi-plot analysis of the PCA

**Fig. 6:**
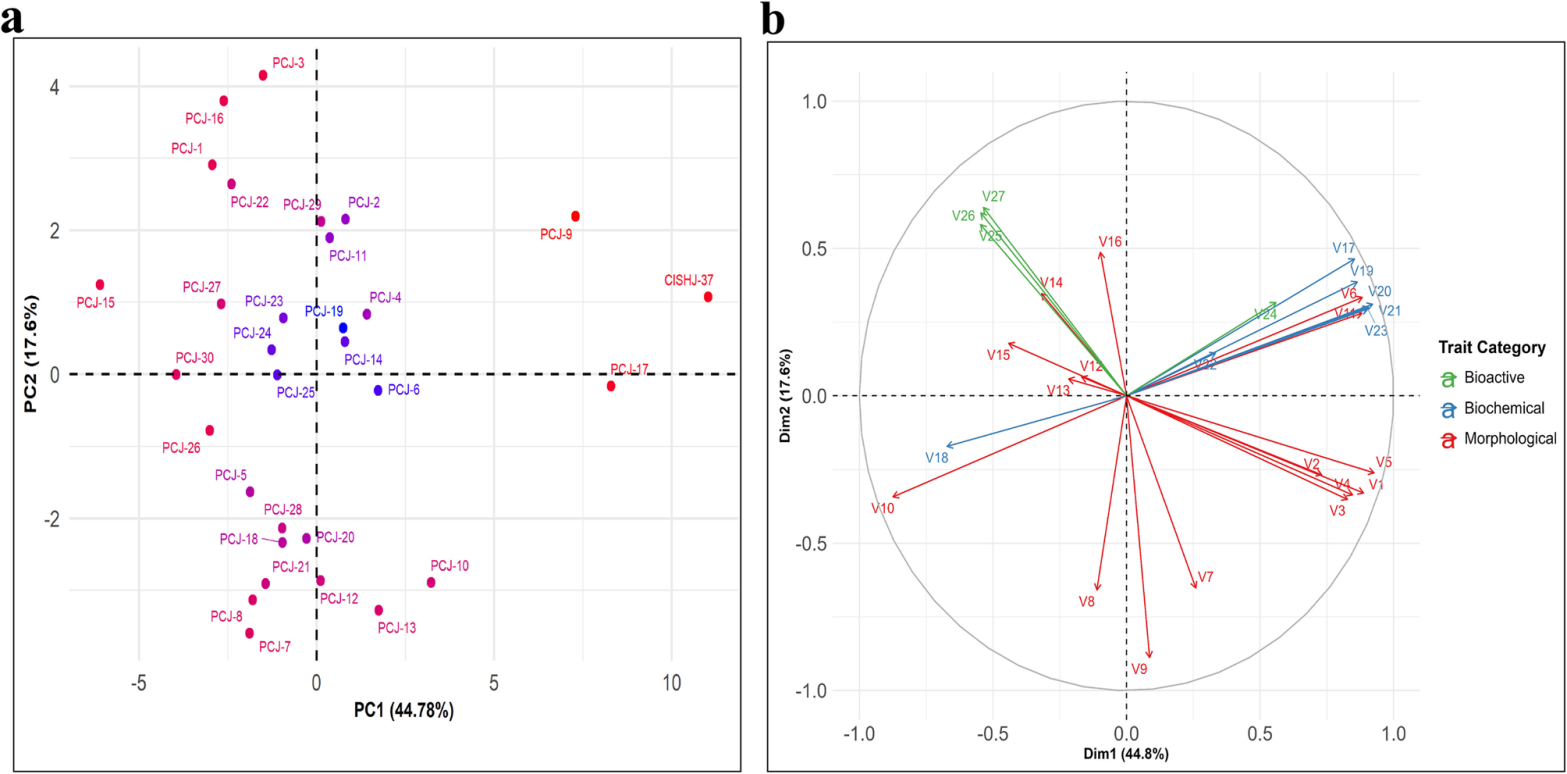
PCA bi-plots based on morpho-biochemical and bioactive traits of *Syzygium cumini (L.)* Skeels genotypes. **a** Distribution of 31 genotypes along PC1 (44.78%) and PC2 (17.6%) showing trait-based variability. **b** Trait loadings of 27 traits grouped as morphological (red), biochemical (blue), and bioactive (green), with arrows indicating their contribution to variability

A bi-plot was constructed using PC1 (44.8%) and PC2 (17.6%), as they shared a major percentage (62.18%) of the total variance. The 31 *Jamun* genotypes (Fig. 6a) and multiple trait categories (Fig. 6b) were distributed throughout all four quadrants of the biplot, confirming high morpho-biochemical and bioactive diversity. Moreover, this analysis effectively revealed trait–genotype relationships. Genotypes such as CISH J-37, PCJ-9 and PCJ-17 were well aligned along the positive axis of PC1, primarily associated with higher pulp content, TSS and sugar-acid ratio, suggesting superior fruit quality traits. In contrast, PCJ-15, PCJ-3 and PCJ-16 genotypes were located in the upper-left quadrant and showed strong associations with antioxidant activity and TA. This implies their potential as bioactive-rich genotypes, albeit possibly with lower palatability due to smaller fruit size and reduced sweetness. Several genotypes, including PCJ-7, PCJ-8 and PCJ-18 etc. were clustered in the lower-left quadrant, indicating negative associations with PC1-contributing attributes such as fruit size, weight and pulp content reflecting inferior fruits morphological profiles. Genotypes near the origin (e.g., PCJ-14, PCJ-19 and PCJ-25) exhibited intermediate trait combinations, lacking extreme values but maintaining balanced trait expressions. The trait vector distribution revealed relationships among traits such as fruit length, width, and pulp weight, forming a tightly clustered group, reflecting strong positive correlations among morphological traits. The positioning of bioactive traits like TPC, TFC and AA along distinct axes confirmed their independent variation, offering scope for simultaneous selection of quality and functional traits. The projection of trait vectors further elucidated trait relationships, morphological traits like fruit length, width and pulp weight were tightly clustered, indicating strong intercorrelation (Fig. 6a and b).

### Cluster analysis of *Jamun *genotypes and traits using a heatmap

**Fig. 7:**
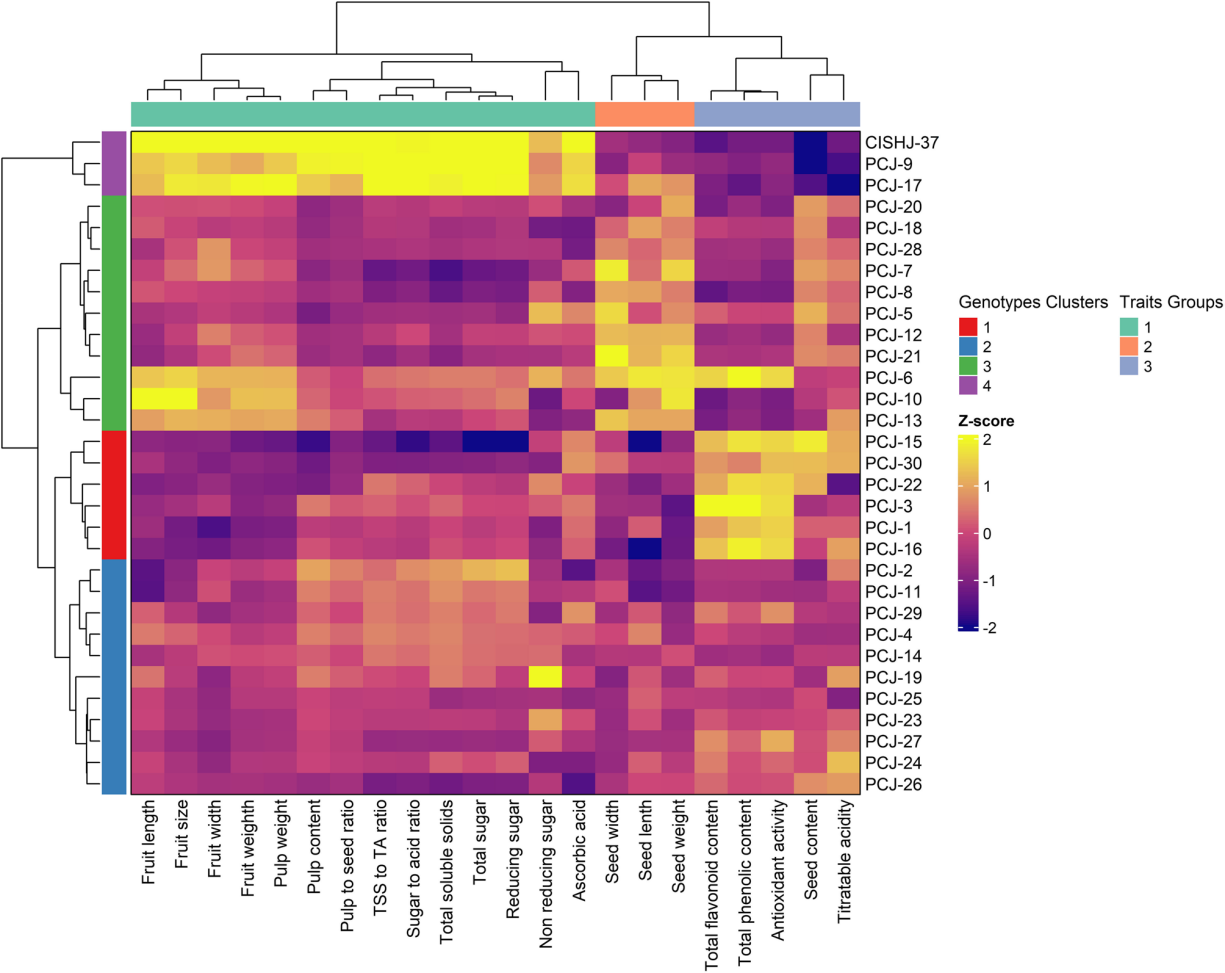
Visualization of clustering patterns of *Syzygium cumini* (L.) Skeels genotypes and quantitative variables based on morpho-biochemical and bioactive characterizations using a heat map. The color scale represents standardized Z-scores, where yellow indicates higher trait values and purple/blue indicates lower values. Rows represent genotypes and columns represent traits. Dendrograms reflect trait and genotype relationships. Colored bars indicate genotype clusters (left) and trait groups (top), identified through clustering analysis

Cluster analysis is employed to arrange a set of observations in a way that groups together individuals who share more similarities with each other than with those who are in separate clusters. Its goal is to identify similar groups within a dataset. However, in this study, a hierarchical clustering heatmap was generated to visualize patterns of variation among 31 *Jamun* genotypes based on 27 morpho-biochemical and bioactive traits (Fig. 7). The Z-score-based color gradient (yellow = high values; purple = low values) revealed clear clustering patterns, indicating high diversity within the studied genotypes. Four major genotype clusters and three main trait groups were identified, reflecting distinct morpho-biochemical and bioactive profiles across the germplasm. The six genotypes (PCJ-15, PCJ-30, PCJ-22, PCJ-3, PCJ-1 and PCJ-16) were clustered in the first cluster (Red), which showed high Z-scores (yellow) for TPC, TFC, antioxidant activity and seed content, but low scores for fruit size-related traits, suggesting their potential as bioactive-rich but smaller-fruited types. Second cluster (light blue) comprising eleven genotypes such as PCJ-2, PCJ-11, PCJ-29, PCJ-4, PCJ-14, PCJ-19, PCJ-25, PCJ-23, PCJ-27, PCJ-24 and PCJ-26. This cluster exhibited intermediate values for key biochemical traits, including pulp content, pulp-to-seed ratio, sugar-to-acid ratio, TSS and total sugars. Cluster 3 (Green) contained eleven genotypes (PCJ-20, PCJ-18, PCJ-28, PCJ-7, PCJ-8, PCJ-5, PCJ-12, PCJ-21, PCJ-6, PCJ-10 and PCJ-13), which had higher values for fruit mass and seed traits, as shown by stronger yellow shades for fruit weight, pulp weight and seed content. Cluster 4 (Purple) was formed by only three superior genotypes, such as CISH J-37, PCJ-9 and PCJ-17, which exhibited consistently high Z-scores across key economic traits, including fruit length, width, weight, pulp content, TSS and sugar-acid ratio (Fig. 7). These genotypes represent elite selections with superior fruit quality and moderate bioactive potential.

## Discussion

### Morphological traits variability

Morphological traits have been proven effective tools for evaluating and characterizing various fruit crops germplasm, such as date palm [35], ber [36], guava [37], cherry [38] and many others. In breeding programs, morphological traits serve as valuable markers for selecting superior genotypes. Morphological characterization is crucial for distinguishing fruit crop varieties, as evident by pomegranate studies, where significant morphological variation was observed among cultivars [24]. Morphology-based selection plays a fundamental role in crop improvement programs aimed at developing genotypes with desirable physical and fruit-related attributes.

In the present study, significant morphological variation was observed among the 31 seedling-origin genotypes, especially in fruit weight, fruit size, pulp content and pulp-to-seed ratio are of commercial importance. Several studies recorded a great array of values for these traits. Anushma and Sane [39] reported fruit weight ranged from 5.58 to 11.18 g, fruit length from 22.50 to 38.70 mm and fruit diameter of 16.30 to 34.20 mm. Devi et al. [20] recorded fruit weights between 1.89 and 16.57 g, fruit length of 17.11 to 43.66 mm and fruit diameter of 11.91 to 39.56 mm in *S. cumini* germplasm from India. Additionally, Khadivi et al. [18] recorded fruit length ranging from 19.38 to 31.16 mm, fruit weight from 2.12 to 8.95 g and fruit diameter from 12.57 to 23.36 mm. The present study recorded comparable results with fruit weight ranging from 5.05 to 22.47 g, fruit length and fruit width, from 2.33 to 4.14 cm and 1.59 to 2.87 cm, respectively. Pulp content, a key determinant for selecting superior genotypes, ranged from 69.43 to 94.49% in this study. Whereas, Singh et al. [40] reported a pulp content range of 79.67 to 86.37% in *Jamun* genotyps from India. Seed-related traits also significantly influence the overall fruit size and weight, with smaller seeds contributing to a higher pulp-to-seed ratio, which is advisable for both fresh consumption and breeding high-quality fruits. Consistent with our findings, Din et al. [41] and Singh et al. [12] recorded pulp-to-seed ratios ranging from 1.31 to 11.33 and 2.8 to 9.5, respectively. The observations suggest that emphasis should be placed on pulp content rather than fruit weight when selecting superior genotypes Devi et al. [20].

Fruit colour and shape serve as important ripening markers and are widely used in cultivar identification [42]. The present study recorded a coefficient of variation (CV) of 41.49% for fruit colour, comparable to the 45.19% reported by Khadivi et al. [18]. The highest variation was obtained in fruit pulp colour, with Khadivi et al. [18] reporting a CV of 48.44% in *Jamun* genotypes from Iran and Din et al. [41] documenting a CV of 46.16% in genotypes from Pakistan. Fruit apex, base and shape are the most important dependent features for fruit characterization [36]. The predominant fruit shape obtained in this study was oblong, consistent with the finding of Khadivi et al. [18]. The predominant fruit skin colour was purple black, while pulp colour was purple white, indicating high anthocyanin content in the *Jamun* fruits [43].

### Variation in biochemical characteristics

Alongside morphological variation, the study revealed significant differences in biochemical traits among genotypes. This study revealed significant variations in commercially important biochemical traits. While some studies have reported TSS exceeding 20 °Brix [12, 41]. The highest TSS recorded in this study was 16.91 °Brix. Such variation can be attributed to genetic differences among genotypes [20]. Observed titratable acidity in the current study aligns with the study conducted by Singh et al. [40] in Indian *Jamun* genotypes. It has been noticed that with an increase in TSS, titratable acidity tends to decrease. This study also recorded significant variations in total sugar content, ascorbic acid content and total phenolic content, aligning with findings by Gajera et al. [44]. In *Tamarindus indica* L., analysis of morpho-physico-chemical variability can lead to the development of better-adapted, higher-yielding and more marketable tamarind varieties [45].

### Diversity in bioactive characteristics

Significant diversity was also seen in bioactive characteristics such as TPC, TFC, ascorbic acid and antioxidant activity. These compounds contribute significantly to the nutraceutical and therapeutic benefits of *Jamun* fruits. El-Safy et al. [46] recorded some bioactive attributes in *Jamun* and found that it contains total phenolics (219.21 mg GAE/100 g), total flavonoids (91.33 mg QE/100 g) and 91.33% antioxidant activity. Bioactive characteristics (total phenols, total flavonoids, ascorbic acid and antioxidant activities etc.) have been used to characterize and select elite germplasm in crops such as *Moringa oleifera*, *Coccinia grandis* and other crops [29, 33, 47, 48]. Moreover, a negative correlation between fruit size and phenolic content was observed in this study, consistent with findings by Gajera et al. [44], who reported that smaller *Jamun* fruits exhibited higher total phenolic content. This inverse relationship may be attributed to the higher sugar concentration in larger fruits, which could dilute phenolic compounds [44]. Positive correlations between secondary metabolites and seed weight of tamarind have also been reported by Singh et al. [45]

### Multivariate insights into trait association and genotype clustering

Strong positive correlations between fruit weight, pulp weight and size-related traits have been found. The significant associations between TSS, sugar content and acidity emphasize the importance of balancing sweetness and acidity for superior consumer acceptance. Din et al. [41] reported a moderate to strong positive correlation among fruit quality traits, further confirming that traits positively correlated with fruit quality and yield directly influence cultivar potential. The correlation of biochemical content with nutritional and medicinal properties has also been highlighted in previous studies [42]. Principal Component Analysis (PCA) was utilized to assess variability patterns among the genotypes. In this study, PC1 was identified as the most significant component (*p* < 0.01) explaining the largest proportion of total variance, primarily driven by fruit size, pulp content and sugar-acid balance. Moreover, PC2 and PC3 (*p* < 0.05) provided further insights into seed characteristics and pulp colour variance, providing secondary classification variables for *Jamun* genotypes. Similar results were in the *Jamun* germplasm from Pakistan [41] and Iraq [18]. In Pakistani *S. cumini* germplasm, PC1 exhibited strong correlations with fruit weight, fruit diameter, seed length, and seed weight, emphasizing the importance of these descriptors in genotype differentiation [41]. The findings of the present study are consistent with research on fruit-bearing trees such as ber [36], persimmon [49], almond [50] and olive [51]. Plant breeders and farmers can use fruit quality and yield-related characteristics (fruit-to-pulp ratio, fruit size, etc.) as target traits because they are highly economically significant [52]. PCA bi-plot analysis has been widely applied to show genotype distribution and similarity other in fruit crops like Olive [53], *Jamun* [18], walnut [22], fig [23] and *Momordica dioica* [48]. The Ward method and Euclidean distance techniques have been frequently applied to data to group related observations and gain a better understanding of the connections between these groups [53]. In a recent study, Mishra et al. [54] used a hierarchical clustering heatmap to demonstrate similarities and differences among physical, biochemical and bioactive compounds in Manila tamarind [*Pithecellobium dulce* (Roxb.) Benth]. Cluster analysis helps in the study of genetic relationships between germplasm by clustering those with similar genetic profiles [45].

### Implications for breeding

The observed variation in morpho-biochemical and bioactive traits provides a valuable foundation for *Jamun* improvement programs. Elite genotypes PCJ-9 and PCJ-17 were identified with superior characteristics, ideal for table purposes for direct consumption. Moreover, genotypes such as PCJ-15, PCJ-30, PCJ-22, PCJ-3, PCJ-1 and PCJ-16, showing greater levels of antioxidant and seed-associated traits, suggest the potential for bioactive traits breeding and nutraceutical applications. Strong positive correlations between fruit weight, pulp weight and size-related traits suggest that larger fruits with a higher edible portion should be selectively targeted for breeding programs. Additionally, the multivariate analyses (PCA and heatmap) provide trait-based grouping that can guide parental selection and hybridization strategies. These insights can guide breeding strategies to develop genotypes suited for both fresh consumption and functional food development.

### Study limitations and future scope

Further multi-location evaluations are necessary to validate genotypes’ performance under diverse environmental conditions. Additionally, molecular validation using DNA-based markers such as Simple Sequence Repeat (SSR) could provide deeper insights. Integrating molecular data with morpho-biochemical traits is suggested to enhance the precision of genotype selection and to support more effective breeding strategies.

## Conclusion

The presented study offers a novel and comprehensive assessment of diversity among seedling-origin *Jamun* genotypes based on morpho-biochemical and bioactive traits. Genotypes PCJ-9 and PCJ-17 demonstrated superior commercial traits such as pulp content, fruit size and sweetness, making them ideal candidates for fresh consumption and direct cultivation. In contrast, six genotypes (PCJ-15, PCJ-30, PCJ-22, PCJ-3, PCJ-1 and PCJ-16) with elevated levels of total phenolics, flavonoids, antioxidant activity and seed content. show potential for breeding programs, targeting bioactive-rich cultivar development, nutraceutical applications and functional food development. Pearson correlation analysis and multivariate approaches such as PCA and heatmap clustering were efficient in classifying genotypes and revealing significant trait links, particularly between fruit weight and pulp content and antioxidant-related characteristics. These findings lay a solid foundation for breeding strategies focused at specific trait improvements. This study also sets the framework for future marker-assisted selection and germplasm conservation strategies in underutilised tropical fruit species such as *Jamun*. However, multi-location studies are required to evaluate genotype performance in diverse environments.

## Supplementary Information

Supplementary Material 1.

## Data Availability

The data that support the findings of this study are available from the corresponding author upon reasonable request.

## Abbreviations

TSS: Total soluble solids
TA: Titratable acidity
TPC: Total phenolic content
TFC: Total flavonoid content
DPPH: 2,2-diphenyl-1-picrylhydrazyl
QE: Quercetin equivalent
GAE: Gallic acid equivalent
PC: Principal Component
