# Supplementary material for: Multivariate assessment of morpho-biochemical and bioactive diversity in Syzygium cumini (L.) Skeels for the selection of superior genotype and breeding applications
Source: BMC Plant Biol. 2025 Jul 26;25:962. doi: 10.1186/s12870-025-06977-x (PMC12297786; doi:10.1186/s12870-025-06977-x)
Supplement: Supplementary file 1 — Supplementary Material 1. [file 12870_2025_6977_MOESM1_ESM.docx]

**Table S1** Detailed information about the longitudes and latitudes of 31 jamun genotypes.

| **Site** | **Genotype** | **Information** | **latitude** | **longitude** |
| --- | --- | --- | --- | --- |
| Location-1 | PCJ-1 | Seedling origin | 28.64051010 | 77.16974820 |
|  | PCJ-2 | Seedling origin | 28.64079330 | 77.16818850 |
|  | PCJ-30 | Seedling origin | 28.64059880 | 77.16891190 |
| Location-2 | PCJ-3 | Seedling origin | 28.64089260 | 77.16689040 |
|  | PCJ-4 | Seedling origin | 28.64071590 | 77.16604070 |
|  | PCJ-5 | Seedling origin | 28.64070790 | 77.16543900 |
|  | PCJ-6 | Seedling origin | 28.64011710 | 77.16475400 |
| Location-3 | PCJ-7 | Seedling origin | 28.63910420 | 77.16301150 |
|  | PCJ-8 | Seedling origin | 28.63865840 | 77.16185110 |
|  | PCJ-12 | Seedling origin | 28.63762700 | 77.16108800 |
|  | PCJ-13 | Seedling origin | 28.63710300 | 77.16106800 |
| Location-4 | PCJ-9 | Seedling origin | 28.64495900 | 77.15882000 |
|  | PCJ-10 | Seedling origin | 28.64595600 | 77.15770400 |
| Location-5 | PCJ-11 | Seedling origin | 28.63717270 | 77.16309700 |
|  | PCJ-14 | Seedling origin | 28.63564700 | 77.16383240 |
|  | PCJ-15 | Seedling origin | 28.63530490 | 77.16373220 |
| Location-6 | PCJ-16 | Seedling origin | 28.63145900 | 77.16514300 |
|  | PCJ-17 | Seedling origin | 28.63242700 | 77.16281690 |
|  | PCJ-29 | Seedling origin | 28.63314600 | 77.16549200 |
| Location-7 | PCJ-18 | Seedling origin | 28.63516050 | 77.16122150 |
|  | PCJ-19 | Seedling origin | 28.63795560 | 77.16014060 |
| Location-8 | PCJ-20 | Seedling origin | 28.63401590 | 77.15229990 |
|  | PCJ-21 | Seedling origin | 28.63382300 | 77.15148900 |
| Location-9 | PCJ-22 | Seedling origin | 28.63284790 | 77.14905820 |
|  | PCJ-23 | Seedling origin | 28.63190800 | 77.15232560 |
| Location-10 | PCJ-24 | Seedling origin | 28.63129140 | 77.15338500 |
|  | PCJ-25 | Seedling origin | 28.63131490 | 77.15401320 |
|  | PCJ-26 | Seedling origin | 28.63115300 | 77.15472030 |
| Location-11 | PCJ-27 | Seedling origin | 28.62994280 | 77.15314920 |
|  | PCJ-28 | Seedling origin | 28.63023110 | 77.15256030 |
|  | CISH-37 | Cultivated type |  |  |

**Table S2** Simple correlations between the quantitative morpho-biochemical and bioactive variables utilized in the studied jamun genotypes.

|  | V1 | V2 | V3 | V4 | V5 | V6 | V7 | V8 | V9 | V10 | V11 |
| --- | --- | --- | --- | --- | --- | --- | --- | --- | --- | --- | --- |
| V1 | 1 |  |  |  |  |  |  |  |  |  |  |
| V2 | 0.799** | 1 |  |  |  |  |  |  |  |  |  |
| V3 | 0.922** | 0.65** | 1 |  |  |  |  |  |  |  |  |
| V4 | 0.932** | 0.92** | 0.89** | 1 |  |  |  |  |  |  |  |
| V5 | 0.991** | 0.806** | 0.914** | 0.937** | 1 |  |  |  |  |  |  |
| V6 | 0.679** | 0.547** | 0.596** | 0.613** | 0.74** | 1 |  |  |  |  |  |
| V7 | 0.405* | 0.474** | 0.336 | 0.458** | 0.392* | 0.02 | 1 |  |  |  |  |
| V8 | 0.179 | -0.087 | 0.306 | 0.111 | 0.124 | -0.319 | 0.5** | 1 |  |  |  |
| V9 | 0.414* | 0.351 | 0.433* | 0.448* | 0.361* | -0.288 | 0.71** | 0.661** | 1 |  |  |
| V10 | -0.654** | -0.535** | -0.576** | -0.6** | -0.722** | -0.997** | -0.034 | 0.326 | 0.286 | 1 |  |
| V11 | 0.75** | 0.568** | 0.671** | 0.652** | 0.78** | 0.935** | -0.071 | -0.262 | -0.281 | -0.91** | 1 |
| V17 | 0.571** | 0.471** | 0.516** | 0.539** | 0.642** | 0.893** | -0.056 | -0.414* | -0.316 | -0.897** | 0.837** |
| V18 | -0.48** | -0.36* | -0.5** | -0.479** | -0.529** | -0.505** | -0.187 | 0.123 | 0.018 | 0.51** | -0.505** |
| V19 | 0.59** | 0.474** | 0.558** | 0.572** | 0.664** | 0.817** | 0.038 | -0.332 | -0.214 | -0.825** | 0.773** |
| V20 | 0.691** | 0.522** | 0.631** | 0.627** | 0.755** | 0.922** | 0.079 | -0.28 | -0.173 | -0.923** | 0.868** |
| V21 | 0.681** | 0.512** | 0.623** | 0.619** | 0.745** | 0.913** | 0.078 | -0.281 | -0.166 | -0.916** | 0.852** |
| V22 | 0.241 | 0.203 | 0.209 | 0.211 | 0.255 | 0.292 | 0.028 | -0.065 | -0.089 | -0.285 | 0.322 |
| V23 | 0.652** | 0.501** | 0.619** | 0.619** | 0.725** | 0.844** | 0.13 | -0.253 | -0.126 | -0.853** | 0.785** |
| V24 | 0.498** | 0.455* | 0.438* | 0.483** | 0.507** | 0.495** | -0.12 | -0.081 | -0.19 | -0.466** | 0.64** |
| V25 | -0.557** | -0.384* | -0.537** | -0.484** | -0.557** | -0.348 | -0.404* | -0.18 | -0.378* | 0.346 | -0.352 |
| V26 | -0.611** | -0.415* | -0.593** | -0.535** | -0.597** | -0.306 | -0.35 | -0.201 | -0.451* | 0.296 | -0.345 |
| V27 | -0.578** | -0.38* | -0.613** | -0.523** | -0.568** | -0.322 | -0.402* | -0.245 | -0.462** | 0.318 | -0.322 |

**Table S 2** (Continued)

|  | V17 | V18 | V19 | V20 | V21 | V22 | V23 | V24 | V25 | V26 | V27 |
| --- | --- | --- | --- | --- | --- | --- | --- | --- | --- | --- | --- |
| V17 | 1 |  |  |  |  |  |  |  |  |  |  |
| V18 | -0.619** | 1 |  |  |  |  |  |  |  |  |  |
| V19 | 0.943** | -0.835** | 1 |  |  |  |  |  |  |  |  |
| V20 | 0.972** | -0.636** | 0.93** | 1 |  |  |  |  |  |  |  |
| V21 | 0.955** | -0.621** | 0.913** | 0.989** | 1 |  |  |  |  |  |  |
| V22 | 0.353 | -0.26 | 0.349 | 0.325 | 0.181 | 1 |  |  |  |  |  |
| V23 | 0.934** | -0.813** | 0.984** | 0.959** | 0.946** | 0.323 | 1 |  |  |  |  |
| V24 | 0.561** | -0.487** | 0.591** | 0.527** | 0.491** | 0.361* | 0.541** | 1 |  |  |  |
| V25 | -0.203 | 0.272 | -0.258 | -0.34 | -0.35 | -0.019 | -0.343 | 0.046 | 1 |  |  |
| V26 | -0.185 | 0.253 | -0.23 | -0.323 | -0.338 | 0.016 | -0.315 | 0.029 | 0.938** | 1 |  |
| V27 | -0.172 | 0.232 | -0.21 | -0.309 | -0.316 | -0.033 | -0.3 | 0.104 | 0.952** | 0.944** | 1 |
